# Role of OATP1B1 and OATP1B3 in Drug-Drug Interactions Mediated by Tyrosine Kinase Inhibitors

**DOI:** 10.3390/pharmaceutics12090856

**Published:** 2020-09-09

**Authors:** Dominique A. Garrison, Zahra Talebi, Eric D. Eisenmann, Alex Sparreboom, Sharyn D. Baker

**Affiliations:** Division of Pharmaceutics and Pharmacology, College of Pharmacy, The Ohio State University, Columbus, OH 43210, USA; garrison.220@osu.edu (D.A.G.); talebi.9@osu.edu (Z.T.); eisenmann.11@osu.edu (E.D.E.)

**Keywords:** OATP1B1, OATP1B3, tyrosine kinase inhibitors, drug-drug interactions

## Abstract

Failure to recognize important features of a drug’s pharmacokinetic characteristics is a key cause of inappropriate dose and schedule selection, and can lead to reduced efficacy and increased rate of adverse drug reactions requiring medical intervention. As oral chemotherapeutic agents, tyrosine kinase inhibitors (TKIs) are particularly prone to cause drug-drug interactions as many drugs in this class are known or suspected to potently inhibit the hepatic uptake transporters OATP1B1 and OATP1B3. In this article, we provide a comprehensive overview of the published literature and publicly-available regulatory documents in this rapidly emerging field. Our findings indicate that, while many TKIs can potentially inhibit the function of OATP1B1 and/or OATP1B3 and cause clinically-relevant drug-drug interactions, there are many inconsistencies between regulatory documents and the published literature. Potential explanations for these discrepant observations are provided in order to assist prescribing clinicians in designing safe and effective polypharmacy regimens, and to provide researchers with insights into refining experimental strategies to further predict and define the translational significance of TKI-mediated drug-drug interactions.

## 1. Introduction

The economic burden of drug-related morbidity and mortality as a result of non-optimized medication therapy is estimated to be more than 16% of total US health care annual expenditures [1]. Overlooking major pharmacokinetic characteristics of a drug is one of the key players in inappropriate pharmaceutical dosing, which can lead to reduced efficacy and an increased rate of adverse drug reactions (ADRs) requiring medical intervention [2]. Pharmacokinetic drug-drug interactions (DDIs) can be responsible for about half of all DDIs depending on the patient group [3,4]. Furthermore, these DDIs have the potential to cause very pronounced (several hundred-fold) and abrupt changes in concentration and effect of the victim drug, depending on the start and stop of the causative (perpetrator) comedication and on fluctuations of its concentration during therapy [2,5,6].

Different components in absorption, distribution, metabolism and excretion can affect the overall pharmacokinetic profile of drugs. For agents that primarily undergo hepatic elimination, transport-mediated mechanisms of hepatocellular uptake can have a particularly significant clinical impact on pharmacotherapy; thus, this field of research has gained increased attention in recent years [7]. The organic anion transporting polypeptides OATP1B1 and OATP1B3 are examples of such transporters that can facilitate the uptake of a diverse array of xenobiotics, including many anticancer drugs, into the liver in advance of metabolism, and that are sensitive to inhibition by other medicines given concurrently.

Two of the most commonly acknowledged risk factors of DDIs are polypharmacy and advanced age [2,8,9,10]. Consistent with this notion, cancer patients are particularly at high risk for the occurrence of potentially harmful DDIs, since they often take a large number of medications concomitantly, which tends to increase as their disease progresses, and because the majority of cancer diagnoses happens in older ages [10,11]. Indeed, prior investigations have demonstrated that as many as 30% of cancer patients receiving chemotherapeutic treatment are at a risk for DDIs [12,13]. As the number of new treatment options in oncology continues to grow, DDIs are increasingly recognized as significant health hazards that can negatively influence treatment outcomes. These issues are particularly concerning given the increasing use orally-administered chemotherapeutic agents. While such drugs offer advantages in terms of patient preference, the convenience of use, reduced healthcare resource utilization, the possibility to achieve sustained drug exposure associated with the need for chronic use without requiring prolonged drug infusions, and may improve the overall quality of life, recent studies have suggested that the use of such agents increases the risk of potentially serious DDIs with commonly used outpatient medications [14]. In addition, unsupervised administration of other medications as well as their possibly prolonged use has been advanced as concerns with oral chemotherapy drugs, which could potentiate DDIs that may remain unanticipated. Although recent studies have suggested that the prevalence of DDIs with oral chemotherapy drugs is as high as 50% with nearly 20% potentially increasing toxicity, the clinical impact of DDIs involving oral chemotherapy remains largely unstudied [10].

In this article, we provide an overview of this field of research in relation to tyrosine kinase inhibitors (TKIs), a rapidly expanding group of orally-administered drugs commonly used in the treatment of solid tumors and hematological malignancies, with particular emphasis on OATP1B1- and OATP1B3-related mechanisms. In addition to reviewing existing published data, we aimed to identify potential knowledge gaps that could help improve our understanding of the clinical impact of DDIs mediated through this mechanism.

## 2. Tyrosine Kinase Inhibitors (TKIs)

Since the US Food and Drug Administration (FDA) approval of the first TKI, imatinib, in 2001 for the treatment of chronic myeloid leukemia (CML), almost 50 additional TKIs have been approved for the treatment of various cancers, and many more are currently being developed and evaluated [15,16]. Protein tyrosine kinases (PTKs) are enzymes that catalyze the transfer of a gamma phosphate group from adenosine triphosphate (ATP) to a tyrosine residue on a protein. The phosphorylation of PTKs leads to the downstream activation of signal transduction pathways that are important in the regulation of cell growth, differentiation, and a series of other physiological and biochemical processes involved in cell survival and migration. Dysregulation of PTK function results in proliferation disorders, with those most notably being cancers [17,18,19]. Because of their importance in signal transduction, many PTKs have been the target of therapeutic intervention with the use of small-molecule TKIs. As a result, TKIs function by competing with ATP for the ATP-binding pocket of PTKs, thus reducing the downstream signaling cascade and provide useful targeted strategies in oncogenic treatment [20,21].

While TKIs have revolutionized anticancer therapy, some challenges have also risen in the use of these agents. Unlike conventional cytotoxic agents that are given intravenously, TKIs are administered orally and daily for prolonged periods [22]. As mentioned before, while this is more convenient, this also increases their susceptibility to unpredictable patterns of oral absorption and causes both wide inter-individual pharmacokinetic variability and potential for DDIs with co-administered agents [23,24,25]. Most TKIs are highly prone to cause DDIs [26], as patients receiving these agents are often subsequently treated for concomitant diseases, and because polypharmacy is highly prevalent [25]. Comorbid conditions such as hypertension, chronic obstructive pulmonary disease, diabetes, cardiovascular disease, congestive heart failure, and peripheral vascular disease are frequently reported in the population of cancer patients [27], and this further increases the risk for potential DDIs. Indeed, a recent study indicated that 97.1% of patients receiving treatment with TKIs were using at least one other drug simultaneously, with a median of 4 concurrent medications, and 47.4% experienced at least one potential TKI-mediated DDI [28]. In another study, 44.7% of the potential DDIs identified involving TKIs were considered severe [29]. Interestingly, most available data in this field have investigated TKIs as victims in DDIs [30,31,32,33], and conclusive information on their role as perpetrators in DDIs is generally lacking.

## 3. Organic Anion Transporting Polypeptides (OATPs)

The vast majority of orally-administered TKIs are eliminated from the body by enzyme-mediated metabolism, which occurs predominantly in the liver, followed by biliary or urinary excretion of the metabolites. These processes require drugs to cross the selectively permeable biological membrane of hepatocytes and are dependent, at least in part, on interaction with membrane transporters. These include the organic anion transporting polypeptides (OATPs), a family of influx transporters expressed in various tissues, including the liver [34,35,36]. Experimental studies with TKIs have predominantly evaluated transport by the liver-specific transporters OATP1B1 and OATP1B3, which are encoded by the *SLCO1B1* and *SLCO1B3* genes [37], respectively. Moreover, it has also been shown that some TKIs can additionally act as inhibitors of the transporters for which they are substrates [38]. Inhibition of OATPs can lead to defective elimination, result in sudden increases in plasma concentration and area under the curve (AUC) for drugs that are substrates of these transporters [36], and ultimately increase the risk of therapy-related side effects. Known substrates of OATP1B1 and OATP1B3 include statins, repaglinide, olmesartan, enalapril, valsartan, several xenobiotic glucuronide metabolites, as well as a host of cytotoxic chemotherapeutic agents, including the taxanes paclitaxel and docetaxel, the platinum-based drug cisplatin, and methotrexate. As hypertension and diabetes are among the prevalent comorbidities in cancer patients, many xenobiotic OATP1B1 and OATP1B3 substrate drugs are likely to be co-administrated with OATP-inhibitory TKIs, and therefore, clinically significant toxicities such as rhabdomyolysis, hyperkalemia, and hypoglycemia can be anticipated [39,40,41].

## 4. Regulatory Guidance Documents

As more and more DDIs involving uptake transporters have been reported in recent years, so have regulatory agencies such as the FDA and the European Medicines Agency (EMA) put increasing emphasis on investigating each new drug entity for their potential to induce/inhibit such transporters. It should be noted that both the “EMA Guideline on the Investigation of Drug Interactions” and “FDA guidance for In Vitro Drug Interaction Studies—Cytochrome P450 Enzyme- and Transporter-Mediated Drug Interactions” recognize the fact that the field of transporter interaction assessments is still rapidly evolving and therefore the recommendations offered are relatively flexible and advocate the use of a variety of methods. However, some specifications have been proposed as a means to ensure that the in vitro models have optimal prediction potential for transporter-mediated interactions:

- Both the FDA and EMA documents suggest that the sponsor should conduct in vitro studies to evaluate whether an investigational drug is an inhibitor of OATP1B1 and/or OATP1B3.
- Both documents recommend using an appropriate, predictive in vitro models, such as human hepatocytes or mammalian cells engineered to overexpress transporters of interest (e.g., CHO, HEK293, MDCK) to explore potential transporter interactions.
- Different concentrations of the investigational drug on the transport of a specific substrate should be investigated, such that at least 3 and 4 concentrations should be tested, according to EMA and FDA guidance documents, respectively, and values for the inhibition constant (Ki) should be obtained, with known inhibitors present as controls.
- According to EMA, Ki values that are lower than a concentration representing 25-fold the unbound hepatic inlet concentration after oral administration warrant the conduct of an in vivo DDI study with the use of a prototypical probe substrate. The most recent FDA guidance, which aligns with the EMA, uses unbound concentrations of the investigational drug, not the total drug, for the calculation of R values with the formula R = 1 + ((fu,p × Iin,max)/ IC_50_) where fu,p is the unbound fraction in plasma, IC_50_ is the half-maximal inhibitory concentration and Iin, max is the estimated maximum plasma inhibitor concentration at the inlet to the liver. An R-value ≥ 1.1 suggests that the drug has the potential to inhibit OATP1B1 and/or OATP1B3 in vivo.
- The 2017 version of the FDA guidance on in vitro assessment of DDIs requires a strategy employing a 30-min preincubation with the inhibitor before the addition of substrate. Although this design is recommended as it may lead to changes in the observed IC_50_ values, the latest version of the guidance does not specify an exact duration of the preincubation conditions.
- The FDA guidance also mentions that the observed degree of inhibition by a particular agent can be dependent on the substrate used in the experiment, and therefore it has been suggested that substrates more likely to be used in clinical studies, or substrates that usually generate lower IC_50_ values for known inhibitors should be chosen in in vitro investigations to avoid underestimation of effects in vivo.

## 5. Identification and Retrieval of Relevant Data

**Figure 1:**
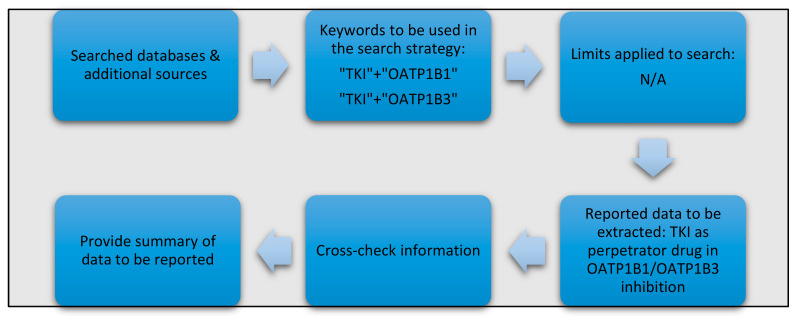
Applied methods for the acquisition of relevant data on TKI-related interactions with OATP1B1 and OATP1B3.

Acquisition of the data for this article was compiled independently up to and including June 2020 by various members of the Division of Pharmaceutics and Pharmacology at the Ohio State University with specific expertise in drug transporters (D.A.G.), pharmacy (Z.T.), and cancer pharmacology (E.D.E.), and subsequently reviewed by members with expertise in pharmacokinetics (A.S.) and TKIs (S.D.B.). Data on FDA-approved TKIs was extracted from the full prescribing information as provided by the respective drug manufacturers. A search was subsequently conducted using publicly-available, unpublished databases from the FDA and EMA guidance documents for industry to further collect information on OATP1B1 and OATP1B3 inhibition studies previously conducted for each of the TKIs (Figure 1). It should be noted that although published studies have indicated that certain TKIs such as erlotinib are inhibitors of 2B1 and can cause DDIs, this was considered beyond the scope of the present article since regulatory guidance documents lack information on this transporter [42].

All DDI data included for consideration focused exclusively on the TKIs as inhibitors of the transporter (the perpetrator) of interest. The selection of relevant literature articles for inclusion was performed based on predefined inclusion/exclusion criteria, where eligible articles included either peer-reviewed publications, meeting abstracts, and previously published reviews. As a primary search module, PubMed (National Library of Medicine) was utilized to identify potentially relevant publications using the following MeSH terms in the search strategy: [“TKI of interest”] AND [OATP1B1] or [“TKI of interest”] AND [OATP1B3]. Google Scholar was consecutively consulted to ensure no published article of relevance to this literature review was omitted. Three authors (D.A.G., Z.T., and E.D.E.) independently reviewed the collected data for eligibility and accuracy. In our analysis, concordant outcomes were defined as those for which the prescribing information, documentation from the FDA and/or EMA, and all the retrieved published literature on a specific TKI were in agreement that the TKI was either an inhibitor or not an inhibitor of OATP1B1 and/or OATP1B3. Outcomes were considered discordant outcomes if the identified reports on a particular TKI regarding its inhibitory properties towards OATP1B1 and/or OATP1B3 were conflicting. All data of relevance was tabulated to highlight such discrepancies (see below).

## 6. Effects of TKIs on the Function of OATP1B1 and OATP1B3

A descriptive summary of the main findings resulting from surveying the available prescribing information (PIs), and FDA and EMA guidance documents are shown in Table 1. The PIs showed that of the 48 FDA-approved TKIs evaluated, 7 (15%) are claimed to be inhibitors of OATP1B1 and 5 (10%) are inhibitors of OATP1B3. In addition, it is reported that of those 48 TKIs, 22 (48%) and 21 (44%) are reported in the PIs to not be inhibitors of OATP1B1 or OATP1B3, respectively. However, it is of note that the PIs for 19 (40%) of the TKIs do not mention whether or not drug interactions with OATP1B1 are of concern, and 22 (46%) do not mention that information for OATP1B3. As shown in Table 1, some inconsistencies were observed for some TKIs between what is reported in the regulatory guidance. Many of the differences can be accounted for by differences in cutoff for IC_50_ values (shown in Supplementary Materials Tables S1–S9).

Next, we conducted a literature search on published data addressing OATP1B1 or OATP1B3 inhibition by different TKIs. In vitro, in vivo, and clinical data were extracted. The details of the articles were inserted into tables (shown in Supplementary Materials Tables S1–S9) [43,44,45,46,47] For alectinib, avapritinib, baracitinib, binimetinib, brigatinib, cobimetinib, dacomitinib, encorafenib, erdafitnib, fedratinib, gilteritinib, ibrutinib, laroctrectinib, lorlatinib, midostaurin, pexidartinib, ponatinib, trametinib, and zanbrutinib no published reports were found. In data collected for 17 TKIs, the results of the published data were largely inconsistent in that some of the published results for a given TKI identified the TKI as an inhibitor of OATP1B1 or OATP1B3, while other sources identified it expressly as a non-inhibitor. It should be noted that different transfected cell lines (Flp-In T-Rex293, HEK293, MDCK-II, CHO, SF9, or HepaRG) and different substrates were used in the various studies. The latter included estradiol-17b-d-glucuronide (E2G), 8-(2-(fluoresceinyl)-aminoethylthio)-adenosine-3′,5′-cyclic monophosphate (8FcA), fluorescein (FL), 2′,7′-dichlorofluorescein (DCF), valsartan, atorvastatin, SN-38, Na-Fluo, fluvastatin, estrone-3-sulfate (E1S) for OATP1B1 and taurocholic acid (TCA), cholecystokinin octapeptide (CCK-8) for OATP1B3. Furthermore, the preincubation time, the method of detection, the data analysis metric (percent inhibition or IC_50_), and even the concentration of the TKI were found to vary among the published reports. The details of these methodological differences are summarized in Table 2.

Data from clinical and in vivo studies were also collected and reviewed for this article, the results of which can be seen in the supplements. Very few studies have directly investigated the role of OATPs in TKI pharmacokinetics with different methodologies, however the results from available studies seem to be consistent with regulatory data. Since the main scope of this review is to focus on discrepancies between published data and FDA and EMA guidelines, their results were not further explored here. Moreover, as OATP1B1 and OATP1B3 substrates used in the retrieved data have complex pharmacokinetic profiles involving drug-metabolizing enzymes and other transporters, the results of such case reports should be carefully analyzed to decide on the importance of each part of the pathway [48,49,50,51,52,53,54,55,56,57,58,59].

### 6.1. Omissions

In numerous studies, TKIs have been indicated as victims in DDIs while considerably less is known about their role as perpetrators via transporter inhibition [32,60,61,62,63,64]. In this context, it is noteworthy that transporter inhibition studies are not required by regulatory agencies for approval, but rather recommended to evaluate DDI potential [38].

Currently, there are 20 FDA approved TKIs for which the PI does not contain any information on their inhibitory effects on OATP1B1 and/or OATP1B3, and this is the case for both agents approved long ago as well as those that were approved more recently. The transport interactions of some of these omitted drugs have been examined by academic investigators as reported in the published literature, and it seems prudent that this information is captured and included in the future in individual PIs and regulatory databases alike. Interestingly, we found that some of the PIs address DDIs that are plausibly attributable to OATPs but this is not always consistently acknowledged due to inconclusive mechanistic insights. For example, dasatinib can dramatically increase plasma levels of the dual OATP1B1 and CYP3A4 substrate, simvastatin, and the individual contribution of each one of these pharmacokinetic components to the DDI is not clearly defined. On the other hand, for many TKIs, no data were found in the published literature on their potential to inhibit OATP1B1 and/or OATP1B3.

### 6.2. Discrepancies

The discrepancies observed during our evaluation can be categorized into two groups: discrepancies between the information provided by EMA and FDA, and discrepancies between different published articles. Table 1 summarizes the cases where data provided by FDA and EMA data were not congruent in terms of reported OATP-inhibitory properties of TKIs. Specific discrepancies of interest are highlighted below. Authors do acknowledge that reporting an IC_50_, even when it is relatively low, does not guarantee a significant clinical impact, unless special formulas are implemented, therefore the inconsistencies reported here, address instances where the guidance is not followed and the reported IC_50_ is not further explored:

- The PI and FDA guidance documents for baricitinib report the agent as an OATP1B3 inhibitor, whereas the EMA documents claim that it is not an inhibitor of this transporter. The existence of this discrepancy is not explained or discussed in any of the regulatory materials.
- For ceritinib, the PI and EMA state that based on in vitro data, the TKI is unlikely to inhibit OATP1B1 and OATP1B3 at clinically-relevant concentrations. However, the FDA guidance document for ceritinib reports that ceritinib inhibits OATP1B1 and OATP1B3 by 31.8% and 24.1%, respectively, and that because the R-value is <1.25, an in vivo study was considered unnecessary. However, the FDA guidance on DDI potential states that a drug has the potential to inhibit OATP1B1 or OATP1B3 in vivo if the R-value is >1.1
- The PI for crizotinib reports the TKI as not an inhibitor of OATP1B1 or OATP1B3, but the FDA guidance reports that crizotinib demonstrated a weak, concentration-dependent inhibitory effect on pravastatin, an OATP1B1 substrate, and rosuvastatin, an OATP1B3 substrate uptake, with IC_50_ values of 48 µM and 44 μM, respectively.
- The PI and FDA documents state that OATP1B1 and OATP1B3 are not inhibited by laroctrectinib, although the EMA materials state that there are inhibitory effects of laroctrectinib on OATP1B1 with an IC_50_ of 48 µM.
- For lenvatinib, the PI states that there is no potential to inhibit OATP1B1 *in vivo*, whereas in the FDA guidance it is concluded that lenvatinib inhibited OATP1B1 with an IC_50_ of 7.29 µM.
- The PI and FDA report that lorlatinib does not inhibit OATP1B1 and OATP1B3, while the EMA claims that this TKI has the potential to inhibit these transporters at clinically-relevant concentrations.
- For osimertinib, the PI and FDA information state that is no observed inhibition of OATP1B1 and OATP1B3, whereas the EMA claims that osimertinib inhibits transport by OATP1B1 and OATP1B3 albeit at concentrations that are unlikely to result in a clinically-significant DDI.

Some of the potential explanations for these discrepancies are similar to those responsible for the apparent discrepant data between different published articles and are discussed in more detail below. However, some interesting points might explain the inconsistencies in regulatory data, such as the equations used to establish whether a clinical evaluation is indeed necessary for the drug or not. While EMA suggests calculating (R = 1 + Iu,in,max/Ki or IC_50_) ≥ 1.04, FDA uses a different equation and different cutoff criteria (R = 1 + Iu,in,max/Ki or IC_50_) ≥ 1.1). This latter equation has been suggested in the latest FDA draft guidance, although prior versions of this document have proposed alternative criteria for consideration. It has also been suggested recently that, while most of the proposed equations and criteria hold merit, they are different in terms of their potential to ultimately arrive at false positive and false negative predictions. In particular, it has been suggested that the equation applied in the EMA guidance has a lower positive predictive value than the one proposed in the current FDA guidance, which offers arguably more dependable predictions [65]. When comparing data from these two regulatory agencies, this aspect should be taken into consideration, along with different manners of data reporting (either with or without calculation of the R-value), and variation in reported IC_50_ values that could be due to differences in the applied methods.

The results of our comparative literature survey also show that there are instances of substantial inconsistency between reports in the published literature as well as between published studies and publicly-available data reported by manufacturers. Since all this collective work is ultimately aimed at improving clinical decision making, it is pertinent to establish an unequivocal, dependable approach to data interpretation. The following are some of the elements that can potentially contribute to the reported inconsistencies:

- Inhibitor concentration: A large number of the published articles have relied on the use of a single concentration of TKI, although regulatory guidance documents specifically recommend the need to perform experiments with at least 3–4 different concentrations, in order to more rigorously evaluate potential inhibitory properties. This is exemplified by a recent study involving the TKIs afatinib, nintedanib, lenvatinib, and ceritinib in which diverse degrees of inhibition were observed depending on the concentration (up to 30 µM), and where some concentrations would even increase transport function [66]. As TKIs tend to get concentrated in the liver and can potentially increase intracellular levels that are much higher than concurrent levels in plasma [2], the selection of relevant concentration ranges to be used in in vitro uptake studies requires careful consideration.
- Data reporting: Several studies have only reported results as percent inhibition relative to control, while more quantitative measures (IC_50_ or inhibition constant) might be more informative and offer increased predictive value. According to regulatory guidance documents, certain equations could be utilized to predict if the observed degree of inhibition has potential clinical relevance. However, such strategies are rarely implemented and reported studies often fail to include positive and negative control inhibitors into the experimental design, which is recommended in the regulatory guidance documents. These issues complicate the interpretation of data and can result in discrepant views on extrapolating from in vitro studies to the clinical situation, as reported for ruxolitinib or crizotinib, where experimental data would suggest statistically significant but not clinically relevant degrees of inhibition [67,68,69].
- Substrate selection: Since substrate-dependent inhibition by xenobiotics, including TKIs, has been well documented and is acknowledged expressly in the FDA guidance document, the degree to which findings obtained with one particular substrate can be extrapolated to other conditions is uncertain, and potentially accounts for several reported inconsistencies. Substrate-dependent inhibition has been previously reported when comparing inhibitory properties in OATP1B1-overexpressed models comparing the substrates fluorescein (FL), 2′,7′-dichlorofluorescein (DCF), atorvastatin, SN-38, and valsartan, as well as in a recent study comparing E2G and 8Fc-A [66], where some TKIs such as lapatinib, pazopanib, and nintedanib show inhibitory effects with some but not all test substrates. The difference between the results for different substrates is occasionally quite substantial; for example, ceritinib can cause 50% inhibition of OATP1B1 function when using FL, DCF, atorvastatin, or SN-38 as test substrates, but causes an apparent increase (by 50%) in OATP1B1-mediated transport of valsartan. Similar results have been reported for nintedanib, which stimulated the OATP1B1-mediated uptake of FL and valsartan, while inhibiting that of DCF and SN-38 (by 70%). One strategy recommended by the FDA to prevent the creation of such apparent, internally conflicting results is to advocate the use of test substrates in the in vitro model system that is predicted to generate the lowest IC_50_ value, or alternatively, to use the most clinically-relevant substrate. While this is a generally useful approach, several published examples highlight the limitations associated with this strategy. For example, Koide et al., have demonstrated that the use of DCF as a model substrate generates the lowest IC_50_ values for most but not necessarily all substrate-inhibitor combinations [66,68] and that TKIs with known OATP1B1-inhibitory properties, such as pazopanib, fail to affect transport function when using the clinically relevant substrates atorvastatin and valsartan [70,71,72,73]. The reported differences in inhibitory properties of TKIs toward the function of transporters such as OATP1B1 as a function of the test substrate used in in vitro studies can directly impact calculated R-values, and influence the reliability of DDI predictions and the clinical decision-making process, especially for weak-to-moderate inhibitors [74].
- Incubation conditions: Several studies have demonstrated that the mechanism by which TKIs inhibit the function of OATP1B1 and/or OATP1B1 can be time-dependent [75], for example in the case of pazopanib, where preincubation times are inversely correlated with the degree of transport inhibition such as that longer preincubation times result in lower IC_50_ estimates [72]. The FDA guidance recommends the inclusion of a preincubation condition, in addition to simultaneous incubation of inhibitor and substrate, to ensure that optimal prediction values can be derived from in vitro experiments. Despite this recommendation, most of the published literature fails to provide specific detail on the design of the reported experiments where the preincubation condition is either not considered or not defined. Although the original FDA guidance recommendation was to include preincubation times of up to 30 min in the experimental study design, recent studies have demonstrated that more prolonged times, for example, one hour in the case of dasatinib or even up to three hours for other compounds, may be required to obtained reliable results [45,76]. Proper consideration of this aspect is especially relevant for a class of agents such as TKIs as they are generally administrated daily for prolonged periods, and may cause transporter inhibition predominantly through an indirect, kinase-mediated mechanism involving post-translational events that affect tyrosine phosphorylation. This suggests that a comprehensive evaluation of TKI-transporter inhibition studies require careful consideration and optimization of preincubation times in order to derive translationally useful DDI predictions.
- Cell line selection: Although regulatory guidance documents do not currently expressly specify any particular cell-based model system for standardized use in in vitro transporter studies, prior findings have supported the notion that the choice of cell lines used for transfection can influence conclusions about inhibitory properties of xenobiotics. Indeed, McFeely et al. have argued that the selection of cell lines as one of the most important factors contributing to variability in observed OATP-mediated transport inhibition when using in vitro models [75]. In addition to intrinsic differences between commonly used cell lines that may be linked with differential baseline expression of other transport mechanisms of putative relevance and artificial compensatory dysregulation of other transporters in overexpressed models, factors such cell origin (e.g., mammalian vs amphibian), cell passage number, cell culture conditions, and maintenance procedures, seeding density, media composition (e.g., presence of binding proteins), and duration of time that cells are in culture (e.g., expression drifting), which are often not clearly documented, could further affect the outcome of each study [77].
- Other contributing variables: In addition to the considerations outlined above as well as in Table 2 and Supplementary Materials Tables S1–S9, several other factors can contribute to variation in the reported transport inhibition data. These include the use of non-standardized software when calculating kinetic parameters such as IC_50_ or Ki, and the implementation of varying methods in quantifying levels of substrate drugs used in the transport assays [77,78]. An example of the latter would the use of an LC-MS/MS-based method to measure the intracellular levels of unchanged substrate drugs, whereas more commonly studies would employ the use of fluorescent substrates of radiolabeled substrates that would be analyzed for total fluorescence or total radioactivity, respectively, and thus would simultaneously measure the total of the parent drug and metabolite(s) formed intracellularly. This is an important methodological difference as certain compounds can undergo rapid enzyme-mediated metabolism once inside cells to form metabolites that may easily escape detection and result in underestimating the actual extent of uptake. Furthermore, even the use of identical protocols in different locales can influence the outcome of particular experimental studies as a result of uncontrollable factors such as interlaboratory differences, as has been documented extensively before for P-glycoprotein IC_50_ determinations [77]. It should also be pointed out that inconsistencies, as reported here for inhibition of OATP1B1- and OATP1B3-mediated transport, are relatively common and have previously been documented for models involving several other drug-metabolizing enzymes and transporters with a putative relevance in predicting clinically relevant DDIs [77,79,80].

## 7. Conclusions

The development and use of TKIs as molecular targeted therapies for the treatment of a diverse array of malignant diseases continues to rapidly increase, and 50 of such agents have now been approved for human use. However, polypharmacy regimens commonly applied in oncology with these TKIs creates a high risk for the occurrence of clinically-relevant DDIs. Although the extent to which such DDIs are influenced by the ability of many TKIs to impact the function of transporter-mediated uptake mechanisms in hepatocytes remains relatively poorly studied, data have accumulated in recent years highlighting that TKIs can act as perpetrators in DDIs by inhibiting OATP1B1 and/or OATP1B3. Many of these recent observations have been made with the use of transfected cell-based in vitro models, and a summary of this available evidence has identified substantial methodological differences between various studies and has highlighted several important limitations in the chosen approaches that have generated incongruent reports. Given that these in vitro studies are the most frequently employed nonclinical tool in aiding decision making for patient care, it is pertinent that regulatory guidance documents and available published literature provide consistent and corresponding results. To further improve consistency in the outcome of transporter-mediated DDI studies involving TKIs, specific recommendations are offered that may assist investigators in the design of future studies in order to provide unequivocal data pertaining to the inhibitory potential of both established as well as investigational TKIs that could be rationally used to further refine the predictive ability of DDIs and ultimately optimize the outcome of treatment in patients with cancer.

## Figures and Tables

**Table 1:** Comparison of regulatory guidance documents on OATP1B inhibition by FDA-approved TKIs.

TKI	Disease Indication	Kinase Target	OATP1B1			OATP1B3		
			**PI**	**FDA**	**EMA**	**PI**	**FDA**	**EMA**
Bacritinib	Rheumatoid Arthritis	JAK	No	No	No	Yes	Yes	No
Ceritinib	Metastatic Non-Small Cell Lung Cancer	ALK	No	Yes	No	No	Yes	No
Crizotinib	Metastatic Non-Small Cell Lung Cancer	ALK, ROS1	No	Yes	-	No	Yes	-
Laroctrectinib	Solid Tumors	NTKR	No	No	Yes	No	No	No
Lenvatinib	Differentiated Thyroid Cancer, Renal Cell Carcinoma, Hepatocellular Carcinoma	VEGFR	No	Yes	Yes	No	No	No
Lorlatinib	Anaplastic Lymphoma Positive Metastatic Non-Small Cell Lung Cancer	ALK	No	No	Yes	No	No	Yes
Midostaurin	Acute Myeloid Leukemia, Aggressive Systemic Mastocytosis, Associated Hematological Neoplasm, Mast Cell Leukemia	FLT3	Yes	Yes	Yes	-	Yes	No
Osimertinib	Metastatic Non-Small Cell Lung Cancer	EGFR	No	No	Yes	No	No	Yes

“Yes” indicates a TKI as an OATP1B1/3 inhibitor provided by the prescribing information, FDA documents, or EMA documents. “No” indicates a TKI is not an inhibitor of OATP1B1/3 inhibitor provided by the prescribing information, FDA documents, or EMA documents. Sources: PI, FDA, EMA documents provided on public databases, details of the links can be found in the Supplementary Materials. Access date: May 2020.

**Table 2:** Inconsistencies in reporting OATP1B inhibition by TKIs in published literature.

TKI	1B1 Inhibitor	Reported Values	1B3 Inhibitor	Reported Values	Model	Pre-Incubation (mins)	Substrate	References
Bosutinib	Yes	>60% inhibition at 10 µM			Flp-In T-Rex293/OATP1B1*1A	15	0.1 mM (3H) (E2G)	FDA: No [68,70,81]
	No	121 ± 6% function remaining after incubation with 10 µM	No	109 ± 5% function remaining after incubation with 10 µM	HEK293/OATP1B1 or 3	UNK	300 nM E3S (1B1) or 2 nM CCK-8 (1B3)	
	Yes	>25% 10 µM on E2G, >50% on 8Fc-A			HEK293/OATP1B1	15	E2G 8Fc-A	
Cabozantinib	No	>15 µM	No	>10 µM	MDCK-II cell monolayers	UNK	OATP1B1: 2 µM; E2G OATP1B3: 2 µM CCK	EMA: No [66,82]
	Yes	59% inhibition at 30 µM			HEK/OATP1B1		3 µM FL	
	Yes	61% inhibition at 30 µM			HEK/OATP1B1		1 µM DCF	
	Yes	74% inhibition at 30 µM			HEK/OATP1B1		1 µM Valsartan	
Ceritinib	Yes	50% inhibition at 30 µM			HEK/OATP1B1	10	3 µM FL	FDA: Yes, PI: No [66]
	Yes	50% inhibition at 30 µM			HEK/OATP1B1	10	1 µM DCF	
	Yes	50% inhibition at 30 µM			HEK/OATP1B1	10	0.5 µM atorvastatin	
	Yes	50% inhibition at 30 µM			HEK/OATP1B1	10	1 µM SN-38	
	No	150% stimulation at 30 µM			HEK/OATP1B1	10	1 µM valsartan	
Crizotinib	No		No		HEK/OATP1B1 or 1B3		11nM (3H]E3S [1B1) 50nM (3H)TCA (1B3) 0.5 µM fluvastatin (1B1) 2 µM fluvastatin (; 1B1)	FDA: Yes, PI: No [67,68]
	Yes	>25% inhibition at 10 µM			HEK293/OATP1B1	15	E2G 8Fc-A	
Erlotinib	No		No		CHO/OATP-1B1 and -1B3	UNK	0.25 μCi/mL (3H)ES (for OATP-1B1) or (3H)CCK-8 (for OATP-1B3)	NI [68,70,81,83]
	Yes	>60% decrease at 10 µM			Flp-In T-Rex293/OATP1B1*1A	15	0.1 mM (3H) (E2G)	
	No	104 ± 5% function remaining after incubation with 10 µM	Yes	50% inhibition at 1.19 µM	HEK293/OATP1B1 or 3	UNK	300 nM E3S (1B1) or 2 nM CCK-8 (1B3)	
	Yes	>25% inhibtion at 10 µM on E2G, >50% inhibition on 8Fc-A			HEK293/OATP1B1	15	E2G 8Fc-A	
Gefitinib	Yes	>70% decrease with 10 µM			Flp-In T-Rex293/OATP1B1*1A	15	0.1 mM (3H) E2G	NI [67,68,70,81]
	Yes	50% inhibition at 17.2 ± 1.47 µM,	Yes	18.8 ± 2.74 mM	HEK293/OATP1B1, OATP1B3,		fluvastatin	
			Inducer	EC50 value of 14.1 ± 4.6 mM	HEK293/ OATP1B1, OATP1B3,		(3H)TCA	
	No	105 ± 3% function remaining after incubation with 10 µM	No	78 ± 3% function remaining after incubation with 10 µM	HEK293/OATP1B1 or 3	UNK	300 nM E3S (1B1) or 2 nM CCK-8 (1B3)	
	Yes	>25% inhibition at 10 µM on E2G, >75% inhibition on 8Fc-A			HEK293/OATP1B1	15	E2G 8Fc-A	
Imatinib	Yes	~20% inhibition at 10 µM			Flp-In T-Rex29/OATP1B1	15	0.1 mM (3H) E2G	NI [47,68,70]
	No				Sf9 /OATP1b1	5	1 µM Na-Fluo	
	Yes	>25% inhibition at 10 µM on both			HEK293/OATP1B1	15	E2G 8Fc-A	
Lapatinib	Yes	>70% inhibition at 10 µM			HEK293/OATP1B1	15	E2G8Fc-A	YES [68,70,81,84,85]
	Yes	>70% inhibition at 10 µM			Flp-In T-Rex29/ OATP1B1	15	0.1 mM (3H) E2G	
	No		Yes, slight inhibition		CHO/ OATP-1B1 or -1B3	UNK	fluro-methotrexate	
	Yes	50% inhibition at 4.0 µM (Sd:2.1)			CHO-OATP1B1	15–30	(3H) E2G	
	No	123 ± 13% function remaining after incubation with 10 µM	No	98 ± 16% function remaining after incubation with 10 µM	HEK293/OATP1B1 or 3	UNK	300 nM E3S (1B1) or 2 nM CCK-8 (1B3)	
Neratinib	No				HEK/OATP1B1	10	3 µM FL	EMA:No [66,81]
	No				HEK/OATP1B1	10	1 µM DCF	
	No				HEK/OATP1B1	10	0.5 µM atorvastatin	
	Yes	30% inhibition at 30 µM			HEK/OATP1B1	10	1 µM SN-38	
	No				HEK/OATP1B1	10	1 µM valsartan	
	No	123 ± 13% function remaining after incubation with 10 µM	Yes	50% inhibition at 18.13 ± 1.21	HEK293/OATP1B1 or 3	UNK	300 nM E3S (1B1) or 2nM CCK-8 (1B3)	
Nilotinib	No				HEK/OATP1B1	10	3 µM FL	NI [66,68,70,83,86,87]
	Yes	~50% inhibition at 30 µM			HEK/OATP1B1	10	1 µM DCF	
	Yes	~50% inhibition at 30 µM			HEK/OATP1B1	10	0.5 µM atorvastatin	
	Yes	~50% inhibition at 30 µM			HEK/OATP1B1	10	1 µM SN-38	
	Yes	~50% inhibition at 30 µM			HEK/OATP1B1	10	1 µM valsartan	
	Yes	>95% inhibition at 10 µM			Flp-In T-Rex29/ OATP1B1	10	0.1 mM (3H) E2G	
	No	110 ± 7% stimulation at 10 µM	No	100 ± 3% function remaining after incubation with 10 µM	HEK293/OATP1B1 or 3	UNK	300 nM E3S (1B1) or 2nM CCK-8 (1B3)	
	Yes	>80% inhibition at 0–20 µM			HEK/OATP1B1		5–40 μM 8Fc-A or 2 μM E2G	
	Yes	50% inhibition at 1.3 μM	Yes		HEK293/OATP1B1 or 3		E2G or 8FcA	
	Yes	>50% at 10 µM, IC_50_: ~1 μM			HEK293/OATP1B1	15	E2G or 8FcA	
	Yes	50% inhibition at 2.78 ± 1.13 μM	No		CHO/ OATP-1B1 or -1B3		0.25 μCi/mL (3H)ES (for OATP-1B1) or (3H)CCK-8 (for OATP-1B3)	
Nintedanib	No	312% stimulation at 30 µM			HEK/OATP1B1		3 µM FL	No [66]
	Yes	74% inhibition at 30 µM			HEK/OATP1B1		1 µM DCF	
	No	133% stimulation at 30 µM			HEK/OATP1B1		1 µM Valsartan	
	Yes	78% inhibition at 30 µM			HEK/OATP1B1		1 μM SN-38	
Pazopanib	No	120% stimulation at 30 µM			HEK/OATP1B1	10	3 µM FL	Yes [66,68,70,71,72,73,83]
	No				HEK/OATP1B1	10	1 µM DCF	
	No				HEK/OATP1B1	10	0.5 µM atorvastatin	
	No				HEK/OATP1B1	10	1 µM SN-38	
	No				HEK/OATP1B1	10	1 µM valsartan	
	Yes	50% inhibition 3.89 ± 1.21 μM	No		CHO/ OATP-1B1 or -1B3		0.25 μCi/mL of (3H)ES (for OATP-1B1) or (3H)CCK-8 (for OATP-1B3)	
	Yes	>50% inhibition with 8Fc-A, >90% inhibition with E2G			HEK293/OATP1B1	15	E2G or (1B1),8FcA (1B1, 1B3)	
	Yes	50% inhibition at 0.79 µM			CHO-OATP1B1	15–30	(3H)-EG	
	No				HEK293/OATP1B1		SN-38	
	Yes	>95% inhibition at 10 µM			Flp-In T-Rex29/OATP1B1	15	0.1 µM (3H) E2G	
	Yes	IC_50_ E1S: 1.42 ± 0.23, IC_50_ E2G: 13.5 ± 6.0			HEK293/OATP1B1	0	(3H) E1S and (3H) E2G	
	Yes	IC_50_ E1S:0.594 ± 0.030 IC_50_ E2G: 7.25 ± 0.53			HEK293/OATP1B2	1	(3H) (E1S) and (3H) E2G	
	Yes	IC_50_ E1S: 0.374 ± 0.074, IC_50_ E2G: 2.58 ± 0.77			HEK293/OATP1B4	30	(3H) E1S and (3H) E2G	
	Yes	IC_50_ E1S: 0.530 ± 0.022, IC_50_ E2G:2.03 ± 0.71			HEK293/OATP1B5	60	(3H) E1S and (3H) E2G	
Regorafenib	No	30% stimulation			HEK293/OATP1B6	10	0.5 µM Atorvastatin	FDA: No [66,68,70,88]
	Yes	50% inhibition at ~10 µM	No		HEK293/OATP1B1/1B3	2	estrone-3-sulfate (1B1)/taurocholic acid (1B3)	
	Yes	>50% inhibition at 10 µM			Flp-In T-Rex29/ OATP1B1	15	0.1mM (3H) E2G	
	Yes	>50% inhibition			HEK293/OATP1B1	15	E2G, 8FcA	
Ruxolitinib	Yes	>25% inhibition at 10 µM on 8Fc-A			HEK293/OATP1B1	15	E2G, 8FcA	No [68,69,70]
	No				HepaRG		4 nM E3S	
	Yes	~20% inhibition at 10 µM			Flp-In T-Rex29/ OATP1B1	15	0.1mM (3H) E2G	
Sorafenib	Yes	>75% at 10 µM on both			HEK293/OATP1B1	15	E2G, 8FcA	NI [66,70,81]
	Yes	>90% inhibition at 10 µM			Flp-In T-Rex293/OATP1B1*1A	15	(3H) E2G 0.1 mM	
	Yes	50% inhibition at 69.6 µM			Flp-In T-Rex293/ OATP1B1*1A	15	0.1 mM (3H) docetaxel	
	No				HEK/OATP1B1	10	3 µM FL	
	No				HEK/OATP1B1	10	1 µM DCF	
	No				HEK/OATP1B1	10	0.5 µM atorvastatin	
	No				HEK/OATP1B1	10	1 µM SN-38	
	No				HEK/OATP1B1	10	1 µM valsartan	
	No	96 ± 7% function remaining after incubation with 10 µM	Yes	68 ± 0.5% function remaining after incubation with 10 µM	HEK293/OATP1B1 or 3	UNK	300nM E3S (1B1) or 2nM CCK-8 (1B3)	
Sunitinib	Yes	>25% decrease at 10 µM			Flp-In T-Rex293/OATP1B1*1A	15	0.1 mM (3H) E2G	NI [68,70,84]
	No	109 ± 10% function remaining after incubation with 10 µM	No	101 ± 10% function remaining after incubation with 10 µM	HEK293/OATP1B1 or 3	UNK	300nM E3S (1B1) or 2nM CCK-8 (1B3)	
	Yes	>25% inhibition			HEK293/OATP1B1	15	E2G, 8FcA	
Vandetanib	Yes	>25% inhibition at 10 µM			Flp-In T-Rex293/ OATP1B1*1A	15	0.1 mM (3H) E2G	NI [53,68,70,83,84]
	No		Yes	50% inhibition at 18.13 ± 1.21	CHO/ OATP-1B1 or -1B3		0.25 μCi/mL of (3H)ES (for OATP-1B1) or (3H)CCK-8 (for OATP-1B3)	
	No	110 ± 6% function remaining after incubation with 10 µM	Yes	71± 5% function remaining after incubation with 10 µM	HEK293/OATP1B1 or 3	UNK	300nM E3S (1B1) or 2nM CCK-8 (1B3)	
	Yes	>25% inhibition at 10 µM			HEK293/OATP1B1	15	E2G, 8FcA	

UNK indicates not mentioned in the study/Unknown. NI is not indicated

## Supplementary Materials

The following are available online at https://www.mdpi.com/1999-4923/12/9/856/s1, Table S1: Prescribing Information, Table S2. FDA guidance for TKI interactions with OATP1B1 and OATP1B3, Table S3. EMA guidance for TKI interactions with OATP1B1 and OATP1B3, Table S4. Comparison of PI, FDA, and EMA regulatory documents, Table S5. In vitro studies for TKIs as perpetrators in OATP-mediated DDIs, Table S6. In vivo studies for TKIs as perpetrators in OATP-mediated DDIs Table S7. Literature on clinical studies focused on TKIs in potential DDIs Table S8. Comparison of the PI, FDA, and EMA guidance documents to the literature for OATP1B1 inhibition and OATP1B3 inhibition, Table S9. TKIs for which no relevant literature was found for OATP-mediated inhibition by TKIs.

## References

[1] Watanabe J.H., McInnis T., Hirsch J.D. (2018). Cost of Prescription Drug–Related Morbidity and Mortality. Ann. Pharmacother..

[2] Fuhr U., Hsin C.-H., Li X., Jabrane W., Sörgel F. (2019). Assessment of Pharmacokinetic Drug–Drug Interactions in Humans: In Vivo Probe Substrates for Drug Metabolism and Drug Transport Revisited. Annu. Rev. Pharmacol. Toxicol..

[3] Magro L., Moretti U., Leone R. (2011). Epidemiology and characteristics of adverse drug reactions caused by drug–drug interactions. Expert Opin. Drug Saf..

[4] Subramanian A., Adhimoolam M., Kannan S. (2018). Study of drug–Drug interactions among the hypertensive patients in a tertiary care teaching hospital. Perspect. Clin. Res..

[5] Backman J.T., Kivistö K.T., Olkkola K.T., Neuvonen P.J. (1998). The area under the plasma concentration-time curve for oral midazolam is 400-fold larger during treatment with itraconazole than with rifampicin. Eur. J. Clin. Pharmacol..

[6] de Jong J., Skee D., Murphy J., Sukbuntherng J., Hellemans P., Smit J., de Vries R., Jiao J.J., Snoeys J., Mannaert E. (2015). Effect of CYP3A perpetrators on ibrutinib exposure in healthy participants. Pharmacol. Res. Perspect..

[7] Gessner A., König J., Fromm M.F. (2019). Clinical Aspects of Transporter-Mediated Drug–Drug Interactions. Clin. Pharmacol. Ther..

[8] Riechelmann R.P., del Giglio A. (2009). Drug interactions in oncology: How common are they?. Ann. Oncol..

[9] Riechelmann R., Girardi D. (2016). Drug interactions in cancer patients: A hidden risk?. J. Res. Pharm. Pr..

[10] Solomon J.M., Ajewole V.B., Schneider A.M., Sharma M., Bernicker E.H. (2019). Evaluation of the prescribing patterns, adverse effects, and drug interactions of oral chemotherapy agents in an outpatient cancer center. J. Oncol. Pharm. Pract..

[11] Howlader N., Mariotto A.B., Besson C., Suneja G., Robien K., Younes N., Engels E.A. (2017). Cancer-specific mortality, cure fraction, and noncancer causes of death among diffuse large B-cell lymphoma patients in the immunochemotherapy era. Cancer.

[12] Riechelmann R.P., Zimmermann C., Chin S.N., Wang L., O’Carroll A., Zarinehbaf S., Krzyzanowska M.K. (2008). Potential Drug Interactions in Cancer Patients Receiving Supportive Care Exclusively. J. Pain Symptom Manag..

[13] van Leeuwen R., Brundel D.H.S., Neef C., van Gelder T., Mathijssen R.H.J., Burger D.M., Jansman F.G.A. (2013). Prevalence of potential drug–drug interactions in cancer patients treated with oral anticancer drugs. Br. J. Cancer.

[14] Bartel S. (2007). Safe practices and financial considerations in using oral chemotherapeutic agents. Am. J. Health Pharm..

[15] Dagher R., Cohen M., Williams G., Rothmann M., Gobburu J., Robbie G., Rahman A., Chen G., Staten A., Griebel D. (2002). Approval summary: Imatinib mesylate in the treatment of metastatic and/or unresectable malignant gastrointestinal stromal tumors. Clin. Cancer Res..

[16] Roskoski R. (2019). Properties of FDA-approved small molecule protein kinase inhibitors. Pharmacol. Res..

[17] Lemmon M.A., Schlessinger J. (2010). Cell Signaling by Receptor Tyrosine Kinases. Cell.

[18] Hubbard S.R., Till J.H. (2000). Protein Tyrosine Kinase Structure and Function. Annu. Rev. Biochem..

[19] Robinson D.R., Wu Y.-M., Lin S.-F. (2000). The protein tyrosine kinase family of the human genome. Oncogene.

[20] Arora A., Scholar E.M. (2005). Role of Tyrosine Kinase Inhibitors in Cancer Therapy. J. Pharmacol. Exp. Ther..

[21] Shawver L.K., Slamon D., Ullrich A. (2002). Smart drugs: Tyrosine kinase inhibitors in cancer therapy. Cancer Cell.

[22] Jeong W., Doroshow J.H., Kummar S. (2013). United States Food and Drug Administration approved oral kinase inhibitors for the treatment of malignancies. Curr. Probl. Cancer.

[23] Herviou P., Thivat E., Richard D., Roche L., Dohou J., Pouget M., Eschalier A., Durando X., Authier N. (2016). Therapeutic drug monitoring and tyrosine kinase inhibitors. Oncol. Lett..

[24] Haouala A., Widmer N., Duchosal M.A., Montemurro M., Buclin T., Decosterd L.A. (2011). Drug interactions with the tyrosine kinase inhibitors imatinib, dasatinib, and nilotinib. Blood.

[25] Iurlo A., Nobili A., Latagliata R., Bucelli C., Castagnetti F., Breccia M., Abruzzese E., Cattaneo D., Fava C., Ferrero D. (2016). Imatinib and polypharmacy in very old patients with chronic myeloid leukemia: Effects on response rate, toxicity and outcome. Oncotarget.

[26] Hussaarts K., Veerman G.D.M., Jansman F.G.A., van Gelder T., Mathijssen R.H.J., van Leeuwen R.W.F. (2019). Clinically relevant drug interactions with multikinase inhibitors: A review. Ther. Adv. Med Oncol..

[27] Fowler H., Belot A., Ellis L., Maringe C., Luque-Fernandez M.A., Njagi E.N., Navani N., Sarfati D., Rachet B. (2020). Comorbidity prevalence among cancer patients: A population-based cohort study of four cancers. BMC Cancer.

[28] Ergun Y., Ozdemir N.Y., Toptas S., Kurtipek A., Eren T., Yazici O., Sendur M.A.N., Akinci B., Ucar G., Oksuzoglu B. (2019). Drug-drug interactions in patients using tyrosine kinase inhibitors: A multicenter retrospective study. J. Buon.

[29] Keller K.L., Franquiz M.J., Duffy A.P., Trovato J.A. (2016). Drug–drug interactions in patients receiving tyrosine kinase inhibitors. J. Oncol. Pharm. Pract..

[30] da Silva C., Honeywell R.J., Dekker H., Peters G.J. (2015). Physicochemical properties of novel protein kinase inhibitors in relation to their substrate specificity for drug transporters. Expert Opin. Drug Metab. Toxicol..

[31] Herbrink M., Nuijen B., Schellens J.H., Beijnen J.H. (2015). Variability in bioavailability of small molecular tyrosine kinase inhibitors. Cancer Treat. Rev..

[32] van Leeuwen R., van Gelder T., Mathijssen R., Jansman F.G.A. (2014). Drug–drug interactions with tyrosine-kinase inhibitors: A clinical perspective. Lancet Oncol..

[33] Schulte R.R., Ho R.H. (2019). Organic Anion Transporting Polypeptides: Emerging Roles in Cancer Pharmacology. Mol. Pharmacol..

[34] Ho R.H., Kim R.B. (2005). Transporters and drug therapy: Implications for drug disposition and disease. Clin. Pharmacol. Ther..

[35] Kalliokoski A., Niemi M. (2009). Impact of OATP transporters on pharmacokinetics. Br. J. Pharmacol..

[36] Shitara Y. (2011). Clinical Importance of OATP1B1 and OATP1B3 in DrugDrug Interactions. Drug Metab. Pharmacokinet..

[37] Zimmerman E.I., Hu S., Roberts J.L., Gibson A.A., Orwick S.J., Li L., Sparreboom A., Baker S. (2013). Contribution of OATP1B1 and OATP1B3 to the disposition of sorafenib and sorafenib-glucuronide. Clin. Cancer Res..

[38] Teo Y.L., Ho H.K., Chan A. (2013). Risk of tyrosine kinase inhibitors-induced hepatotoxicity in cancer patients: A meta-analysis. Cancer Treat. Rev..

[39] Kellick K. (2017). Organic Ion Transporters and Statin Drug Interactions. Curr. Atheroscler. Rep..

[40] Alam K., Crowe A., Wang X., Zhang P., Ding K., Li L., Yue W. (2018). Regulation of Organic Anion Transporting Polypeptides (OATP) 1B1- and OATP1B3-Mediated Transport: An Updated Review in the Context of OATP-Mediated Drug-Drug Interactions. Int. J. Mol. Sci..

[41] Lancaster C.S., Sprowl J.A., Walker A.L., Hu S., Gibson A.A., Sparreboom A. (2013). Modulation of OATP1B-type transporter function alters cellular uptake and disposition of platinum chemotherapeutics. Mol. Cancer Ther..

[42] Chen M., Hu S., Li Y., Gibson A.A., Fu Q., Baker S.D., Sparreboom A. (2020). Role of Oatp2b1 in Drug Absorption and Drug-Drug Interactions. Drug Metab. Dispos..

[43] Podoll T., Pearson P.G., Evarts J., Ingallinera T., Sun H., Byard S., Fretland A.J., Slatter J.G. (2019). Abstract 13: Structure elucidation, metabolism, and drug interaction potential of ACP-5862, an active, major, circulating metabolite of acalabrutinib. Cancer Res..

[44] Ellens H., Johnson M., Lawrence S.K., Chen L., Richards-Peterson L.E., Watson C. (2017). Prediction of the Transporter-Mediated Drug-Drug Interaction Potential of Dabrafenib and Its Major Circulating Metabolites. Drug Metab. Dispos..

[45] Pahwa S., Alam K., Crowe A., Farasyn T., Neuhoff S., Hatley O., Ding K., Yue W. (2017). Pretreatment With Rifampicin and Tyrosine Kinase Inhibitor Dasatinib Potentiates the Inhibitory Effects Toward OATP1B1- and OATP1B3-Mediated Transport. J. Pharm. Sci..

[46] Elsby R., Martin P., Surry D., Sharma P., Fenner K. (2015). Solitary Inhibition of the Breast Cancer Resistance Protein Efflux Transporter Results in a Clinically Significant Drug-Drug Interaction with Rosuvastatin by Causing up to a 2-Fold Increase in Statin Exposure. Drug Metab. Dispos..

[47] Patik I., Kovacsics D., Német O., Gera M., Várady G., Stieger B., Hagenbuch B., Szakács G., Özvegy-Laczka C. (2015). Functional expression of the 11 human Organic Anion Transporting Polypeptides in insect cells reveals that sodium fluorescein is a general OATP substrate. Biochem. Pharmacol..

[48] Bergman E., Hedeland M., Bondesson U., Lennernäs H. (2010). The effect of acute administration of rifampicin and imatinib on the enterohepatic transport of rosuvastatinin vivo. Xenobiotica.

[49] Nakamura Y., Hirokawa Y., Kitamura S., Yamasaki W., Arihiro K., Tatsugami F., Iida M., Kakizawa H., Date S., Awai K. (2013). Effect of lapatinib on hepatic parenchymal enhancement on gadoxetate disodium (EOB)-enhanced MRI scans of the rat liver. Jpn. J. Radiol..

[50] Martin P.D., Gillen M., Ritter J., Mathews D., Brealey C., Surry D., Oliver S., Holmes V., Severin P., Elsby R. (2016). Effects of Fostamatinib on the Pharmacokinetics of Oral Contraceptive, Warfarin, and the Statins Rosuvastatin and Simvastatin: Results From Phase I Clinical Studies. Drugs R&D.

[51] Harvey R.D., Aransay N.R., Isambert N., Lee J.-S., Arkenau T., Vansteenkiste J., Dickinson P.A., Bui K., Weilert D., So K. (2018). Effect of multiple-dose osimertinib on the pharmacokinetics of simvastatin and rosuvastatin. Br. J. Clin. Pharmacol..

[52] Vishwanathan K., Cantarini M., So K., Masson E., Fetterolf J., Ramalingam S.S., Harvey R.D. (2019). Impact of Disease and Treatment Response in Drug–Drug Interaction Studies: Osimertinib and Simvastatin in Advanced Non-Small Cell Lung Cancer. Clin. Transl. Sci..

[53] Calvo E., Lee J.-S., Kim S.-W., Moreno V., Carpeno J.D., Weilert D., Laus G., Mann H., Vishwanathan K. (2019). Modulation of Fexofenadine Pharmacokinetics by Osimertinib in Patients With Advanced EGFR-Mutated Non–Small Cell Lung Cancer. J. Clin. Pharmacol..

[54] Reddy V.P., Walker M., Sharma P., Ballard P., Vishwanathan K. (2018). Development, Verification, and Prediction of Osimertinib Drug-Drug Interactions Using PBPK Modeling Approach to Inform Drug Label. CPT Pharmacomet. Syst. Pharmacol..

[55] Komatsu H., Enomoto M., Shiraishi H., Morita Y., Hashimoto D., Nakayama S., Funakoshi S., Hirano S., Terada Y., Miyamura M. (2020). Severe hypoglycemia caused by a small dose of repaglinide and concurrent use of nilotinib and febuxostat in a patient with type 2 diabetes. Diabetol. Int..

[56] Kendra K., Plummer R., Salgia R., O’Brien M.E.R., Paul E.M., Suttle A.B., Compton N., Xu C.-F., Ottesen L.H., Villalona-Calero M.A. (2014). A Multicenter Phase I Study of Pazopanib in Combination with Paclitaxel in First-Line Treatment of Patients with Advanced Solid Tumors. Mol. Cancer Ther..

[57] Poje D.K., Božina N., Šimičević L., Žabić I. (2020). Severe hyperglycaemia following pazopanib treatment: The role of drug-drug-gene interactions in a patient with metastatic renal cell carcinoma—A case report. J. Clin. Pharm. Ther..

[58] Hamberg P., Mathijssen R.H.J., de Bruijn P., Leonowens C., van der Biessen D., Eskens F.A.L.M., Sleijfer S., Verweij J., de Jonge M.J.A. (2014). Impact of pazopanib on docetaxel exposure: Results of a phase I combination study with two different docetaxel schedules. Cancer Chemother. Pharmacol..

[59] Xu C.-F., Xue Z., Bing N., King K.S., McCann L.A., de Souza P.L., Goodman V.L., Spraggs C.F., Mooser V.E., Pandite L.N. (2012). Concomitant use of pazopanib and simvastatin increases the risk of transaminase elevations in patients with cancer. Ann. Oncol..

[60] Mandery K., Glaeser H., Fromm M.F. (2011). Interaction of innovative small molecule drugs used for cancer therapy with drug transporters. Br. J. Pharmacol..

[61] Lawrence S.K., Nguyen D., Bowen C., Richards-Peterson L., Skordos K.W. (2014). The Metabolic Drug-Drug Interaction Profile of Dabrafenib: In Vitro Investigations and Quantitative Extrapolation of the P450-Mediated DDI Risk. Drug Metab. Dispos..

[62] Filppula A., Neuvonen P.J., Backman J.T. (2014). In Vitro Assessment of Time-Dependent Inhibitory Effects on CYP2C8 and CYP3A Activity by Fourteen Protein Kinase Inhibitors. Drug Metab. Dispos..

[63] Grenader T., Gipps M., Shavit L., Gabizon A. (2007). Significant drug interaction: Phenytoin toxicity due to erlotinib. Lung Cancer.

[64] Kuhn E.L., Lévêque D., Lioure B., Gourieux B., Bilbault P. (2016). Adverse event potentially due to an interaction between ibrutinib and verapamil: A case report. J. Clin. Pharm. Ther..

[65] Vaidyanathan J., Yoshida K., Arya V., Zhang L. (2016). Comparing Various In Vitro Prediction Criteria to Assess the Potential of a New Molecular Entity to Inhibit Organic Anion Transporting Polypeptide 1B1. J. Clin. Pharmacol..

[66] Koide H., Tsujimoto M., Takeuchi A., Tanaka M., Ikegami Y., Tagami M., Abe S., Hashimoto M., Minegaki T., Nishiguchi K. (2017). Substrate-dependent effects of molecular-targeted anticancer agents on activity of organic anion transporting polypeptide 1B1. Xenobiotica.

[67] Sato T., Ito H., Hirata A., Abe T., Mano N., Yamaguchi H. (2017). Interactions of crizotinib and gefitinib with organic anion-transporting polypeptides (OATP)1B1, OATP1B3 and OATP2B1: Gefitinib shows contradictory interaction with OATP1B3. Xenobiotica.

[68] Leblanc A.F., Sprowl J.A., Alberti P., Chiorazzi A., Arnold W.D., Gibson A.A., Hong K.W., Pioso M.S., Chen M., Huang K.M. (2018). OATP1B2 deficiency protects against paclitaxel-induced neurotoxicity. J. Clin. Investig..

[69] Febvre-James M., Bruyère A., Le Vée M., Fardel O. (2018). The JAK1/2 Inhibitor Ruxolitinib Reverses Interleukin-6-Mediated Suppression of Drug-Detoxifying Proteins in Cultured Human Hepatocytes. Drug Metab. Dispos..

[70] Hu S., Mathijssen R.H.J., de Bruijn P., Baker S., Sparreboom A. (2014). Inhibition of OATP1B1 by tyrosine kinase inhibitors: In vitro–in vivo correlations. Br. J. Cancer.

[71] Iwase M., Fujita K.-I., Nishimura Y., Seba N., Masuo Y., Ishida H., Kato Y., Kiuchi Y. (2019). Pazopanib interacts with irinotecan by inhibiting UGT1A1-mediated glucuronidation, but not OATP1B1-mediated hepatic uptake, of an active metabolite SN-38. Cancer Chemother. Pharmacol..

[72] Taguchi T., Masuo Y., Sakai Y., Kato Y. (2019). Short-lasting inhibition of hepatic uptake transporter OATP1B1 by tyrosine kinase inhibitor pazopanib. Drug Metab. Pharmacokinet..

[73] Xu C.F., Reck B.H., Xue Z., Huang L., Baker K.L., Chen M., Chen E.P., Ellens H.E., Mooser V.E., Cardon L.R. (2010). Pazopanib-induced hyperbilirubinemia is associated with Gilbert’s syndrome UGT1A1 polymorphism. Br. J. Cancer.

[74] Izumi S., Nozaki Y., Maeda K., Komori T., Takenaka O., Kusuhara H., Sugiyama Y. (2014). Investigation of the Impact of Substrate Selection on In Vitro Organic Anion Transporting Polypeptide 1B1 Inhibition Profiles for the Prediction of Drug-Drug Interactions. Drug Metab. Dispos..

[75] McFeely S.J., Ritchie T.K., Ragueneau-Majlessi I. (2020). Variability in In Vitro OATP1B1/1B3 Inhibition Data: Impact of Incubation Conditions on Variability and Subsequent Drug Interaction Predictions. Clin. Transl. Sci..

[76] Tátrai P., Schweigler P., Poller B., Domange N., de Wilde R., Hanna I., Gaborik Z., Huth F. (2019). A Systematic In Vitro Investigation of the Inhibitor Preincubation Effect on Multiple Classes of Clinically Relevant Transporters. Drug Metab. Dispos..

[77] Bentz J., O’Connor M.P., Bednarczyk D., Coleman J., Lee C., Palm J., Pak Y.A., Perloff E.S., Reyner E., Balimane P. (2013). Variability in P-Glycoprotein Inhibitory Potency (IC50) Using Various in Vitro Experimental Systems: Implications for Universal Digoxin Drug-Drug Interaction Risk Assessment Decision Criteria. Drug Metab. Dispos..

[78] Volpe D.A., Hamed S.S., Zhang L.K. (2013). Use of Different Parameters and Equations for Calculation of IC50 Values in Efflux Assays: Potential Sources of Variability in IC50 Determination. AAPS J..

[79] Greenblatt D.J., Venkatakrishnan K., Harmatz J.S., Parent S.J., von Moltke L.L. (2010). Sources of variability in ketoconazole inhibition of human cytochrome P450 3Ain vitro. Xenobiotica.

[80] Huang K.M., Uddin M.E., Digiacomo D., Lustberg M.B., Hu S., Sparreboom A. (2020). Role of SLC transporters in toxicity induced by anticancer drugs. Expert Opin. Drug Metab. Toxicol..

[81] Johnston R.A., Rawling T., Chan T., Zhou F., Murray M. (2014). Selective Inhibition of Human Solute Carrier Transporters by Multikinase Inhibitors. Drug Metab. Dispos..

[82] Lacy S., Hsu B., Miles D., Aftab D., Wang R., Nguyen L. (2015). Metabolism and Disposition of Cabozantinib in Healthy Male Volunteers and Pharmacologic Characterization of Its Major Metabolites. Drug Metab. Dispos..

[83] Khurana V., Minocha M., Pal D., Mitra A.K. (2014). Inhibition of OATP-1B1 and OATP-1B3 by tyrosine kinase inhibitors. Drug Metab. Drug Interact..

[84] Kotsampasakou E., Brenner S., Jaeger W., Ecker G.F. (2015). Identification of Novel Inhibitors of Organic Anion Transporting Polypeptides 1B1 and 1B3 (OATP1B1 and OATP1B3) Using a Consensus Vote of Six Classification Models. Mol. Pharm..

[85] Polli J.W., Humphreys J.E., Harmon K.A., Castellino S., O’mara M.J., Olson K.L., John-Williams L.S., Koch K.M., Serabjit-Singh C.J. (2008). The role of efflux and uptake transporters in [N-{3-chloro-4-[(3-fluorobenzyl)oxy]phenyl}-6-[5-({[2-(methylsulfonyl)ethyl]amino}methyl)-2-furyl]-4-quinazolinamine (GW572016, lapatinib) disposition and drug interactions. Drug Metab. Dispos..

[86] Hayden E.R. (2020). Phosphorylation and function of OATP1B1 with tyrosine kinase inhibitors. FASEB J..

[87] Sprowl J.A., Chen M., Gibson A.A., Pasquariello K.Z., Sparreboom A., Hu S. (2019). Characterization of OATP1B1 and OATP1B3 inhibition by Nilotinib. FASEB J..

[88] Ohya H., Shibayama Y., Ogura J., Narumi K., Kobayashi M., Iseki K. (2015). Regorafenib Is Transported by the Organic Anion Transporter 1B1 and the Multidrug Resistance Protein 2. Biol. Pharm. Bull..

